# Epithelioid Hemangioendothelioma in the Tongue: A Rare Case Report

**DOI:** 10.5146/tjpath.2021.01560

**Published:** 2023-01-15

**Authors:** Deniz Surmeli Cirkin, Ilke Evrim Secinti, Esin Dogan, Gul Soylu Ozler

**Affiliations:** Department of Pathology, Hatay Mustafa Kemal University, Faculty of Medicine, Hatay, Turkey; Department of Otorhinolaryngology, Hatay Mustafa Kemal University, Faculty of Medicine, Hatay, Turkey

**Keywords:** Hemangioendothelioma, Tongue, Oral cavity

## Abstract

Epithelioid hemangioendothelioma is a rare malignant vascular neoplasm caused by the proliferation of neoplastic endothelial cells. Epithelioid hemangioendothelioma may develop in any organ, but it is commonly observed in the extremities. The tongue is a very unusual location for epithelioid hemangioendothelioma.

A 55-year-old male patient presented to the outpatient head and neck clinic with lumps in the tongue, pain, and limitation of motion. The polypoid mass detected in the anterior midline of the tongue was excised. Microscopically, the tumor cells included slightly pleomorphic oval or round vesicular nuclei with an eosinophilic cytoplasm that variably contained vacuoles. There were 4 mitoses per 10 high power fields and there was no necrosis. In immunohistochemical study, the tumor cells were positively stained with CD31 and CD34 whereas they were negatively stained with TFE3, SMA, S-100, HHV-8 and EMA. The patient was diagnosed with “epitheloid hemangioendothelioma”.

Only ten cases have been reported in the tongue in the literature. Our case was the eleventh case, and we aimed to report this case as a rare entity with an unusual location.

## INTRODUCTION

Enzinger and Weiss have classified hemangioendothelioma lesions as epithelioid, kaposiform, hobnail (dabska-retiform), composite and epithelioid sarcoma-like heman-gioendothelioma (1). Epithelioid hemangioendothelioma (EHE) is a rare malignant vascular neoplasm caused by the proliferation of neoplastic endothelial cells (2). Epithelioid hemangioendothelioma commonly develops in the extremities, but it may be also seen in any organ such as the liver, breast, lungs, and long bones (3). It is rarely seen in the head and neck including the submandibular region, parotid gland, and oral cavity (4,5). Only 31 cases have been reported in the oral cavity in the literature (6). The gingiva is the most common location in the oral cavity, followed by the tongue, and the maxillary and buccal mucosa, respectively (6,7). Only ten cases of epithelioid hemangioendothelioma of the tongue have been reported in the literature (6).

EHE is driven by WWTR1-CAMTA1 or YAP1-TFE3 fusion genes (8,9). EHEs that occur due to WWTR1-CAMTA1 fusion gene express CAMTA1 while those occurring due to YAP1-TFE3 fuse gene express TFE3 (less specifically). WWTR1-CAMTA1 fusion is positive in 90% of hemangioendothelioma cases (8). Microscopically; endothelial cell proliferation with round nuclei and eosinophilic cytoplasm are observed. There is frequent cytoplasmic vacuolization. These vacuoles vary in size, but are occasionally large blister-like structures that disrupt the shape of the cell (1). This is characterized by proliferating layers, cords, and islands of eosinophilic round or oval-shaped epithelioid cells intertwining with spindle cell threads around proliferating small blood capillaries (10). Tumor cells express positivity with endothelial markers such as CD31, CD34, ERG, and von Willebrand factor (VWF). VWF provides high specificity with cytoplasmic labeling while CD31 and CD34 are more sensitive (11)**.**

## CASE

**Figure 1:**
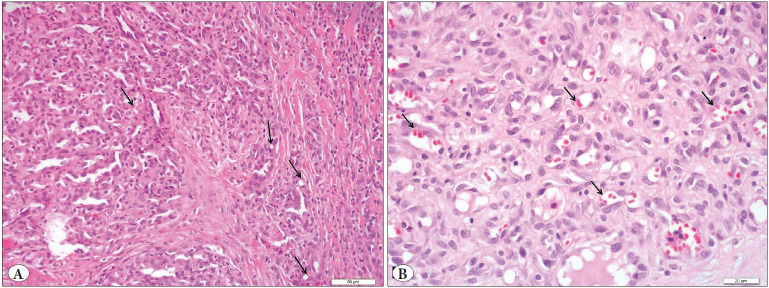
Tumor cells including slightly pleomorphic oval or round vesicular nuclei and cytoplasmic vacuoles (black arrow), H&E x 200 **(A).** Numerous erythrocytes within some vacuoles (arrow head), H&E x 400 **(B).**

**Figure 2:**
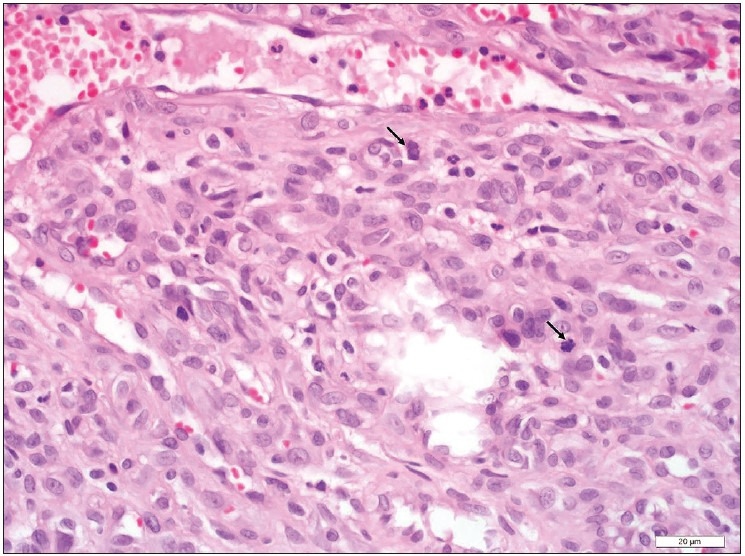
There are two mitoses in one high power field (black arrow), H&E x 400.

**Figure 3:**
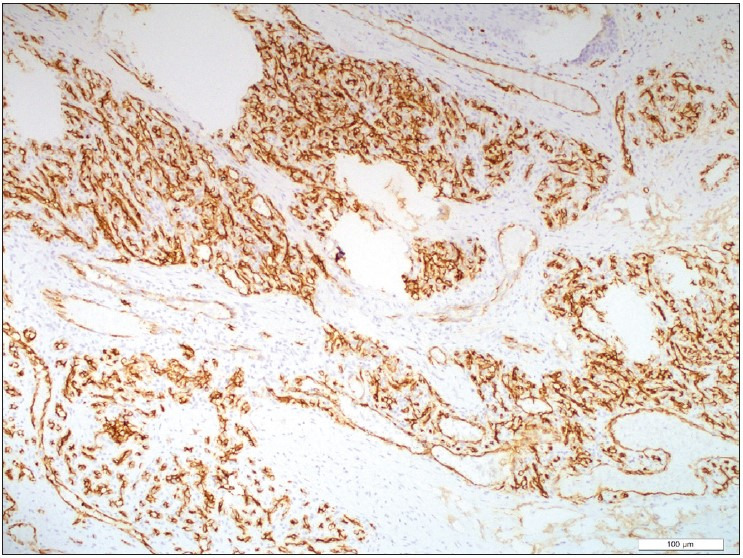
Tumor cells immunoreactive with CD34, CD34 antibody, x100.

**Figure 4:**
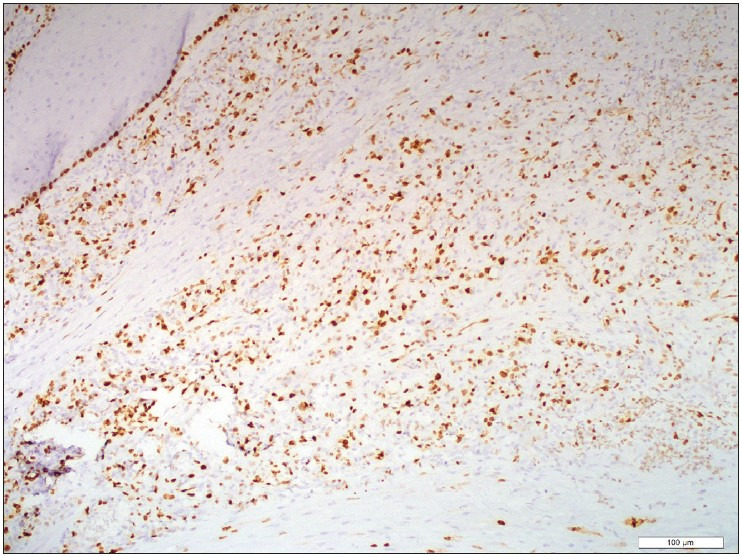
Tumor cells with high proliferative index, Ki67 antibody, x100.

A 55-year-old male patient presented to the outpatient head and neck clinic with lumps in the tongue, pain, and limitation of motion. A polypoid lesion was detected in the anterior midline of the tongue. There was no pathological finding in the laboratory tests. The mass was completely excised and the tissue was sent to the Department of Pathology for histopathologic evaluation. Macroscopically, a 1.4x1x0.3 cm gray-white colored polypoid mass was observed. The sectional surface was observed to have a brown-gray colored hemorrhagic appearance. Microscopically, a tissue sample with non-keratinized acanthotic epithelium was containing an ulcer. A tumor with an infiltrative border was encountered under the ulcer. The tumor consisted of cells with slightly pleomorphic oval or round vesicular nuclei in the fibrous stroma and eosinophilic cytoplasm that variably contained vacuoles (Figure 1). Numerous erythrocytes were found both in the vacuoles and as extravasated (Figure 1). There were 4 mitoses per 10 high power fields and there was no necrosis (Figure 2). On immunohistochemistry, tumor cells were stained positively with CD34 (diluted 1:400, Thermo Scientific, Fremont, CA) and CD31 (diluted 1:100, Thermo Scientific, Fremont, CA) and they were negatively stained with TFE3 (diluted 1:50, ZETA Corporation, California, USA), cytokeratin (diluted 1:100, Thermo Scientific, Fremont, CA) (Figure 3), SMA (diluted 1:800, Thermo Scientific, Fremont, CA), EMA (diluted 1:600, Thermo Scientific, Fremont, CA), S-100 (diluted 1:150, Thermo Scientific, Fremont, CA), and HHV-8 (diluted 1:100, Thermo Scientific, Fremont, CA), Ki67 proliferation index (diluted1:150, Thermo Scientific, Fremont, CA) was 30% (Figure 4). The patient was evaluated as epithelioid hemangioendothelioma based on the histomorphological and immunohistochemical evidence.

## DISCUSSION

Epithelioid hemangioendothelioma was described as a bone and soft tissue vascular tumor, a subtype of hemangioendothelioma by Weiss and Enzinger in 1982 (12). Epithelioid hemangioendothelioma is usually localized in soft tissues and extremities. It may be also localized in the head and neck (13). Epithelioid hemangioendothelioma rarely occurs in the oral cavity and only 31 cases have been reported in the literature until the present day. The gingiva is the most common location in the oral cavity, followed by the tongue, and the maxillary and buccal mucosa, respectively (6,7). Epithelioid hemangioendothelioma may be seen at any age. However, it is more commonly observed in the middle ages and affects both genders approximately at equal rates (1). Clinically, it usually occurs as a painful mass (2). Our case was a 55-year-old male patient who presented to the clinic with a painless mass.

Neoplastic endothelial cell proliferation, eosinophilic vacuolated cytoplasm, and occasionally fusiform cells are observed as the characteristic microscopic features (13). In most cases, there is almost no mitotic activity in the tumor. The microscopic differential diagnosis of epithelioid hemangioendothelioma is performed by taking carcinoma, epithelioid angiosarcoma, melanoma, and angioleiomyoma into consideration (8,13). Epithelioid hemangioendothelioma may be confused with carcinoma with its high cellularity and mitotic activity (12). Immunohistochemically, hemangioendothelioma cells are positive for CD34, CD31, and vonWillebrand factor (14). Positive staining with endothelial markers such as CD31, CD34, and Factor VIII may be helpful in differentiating epithelioid hemangioendothelioma from carcinoma (15). In addition, cytokeratin is strongly positive in almost all carcinomas as well as epithelioid angiosarcomas and epithelioid sarcomas. However, approximately 25% of epithelioid hemangioendotheliomas are cytokeratin and EMA positive, but this is weak and focally positive compared with epithelioid sarcoma (2). Epithelioid angiosarcoma is expected to be strongly positively stained with CD31, CD34, panCK, and EMA during immunohistochemical examination. In our case, tumor cells were stained positive with CD31 and CD34 whereas they were negatively stained with panCK and EMA as the evidence of differentiation from carcinoma, epithelioid sarcoma, and epitheloid angiosarcoma. Melanoma was ruled out since it was S100 negative. Approximately, one fourth of the epithelioid hemangioendothelioma cases show mitotic activity (more than 1 mitosis at 10 high-power fields), significant atypia, and necrosis (1). Cellular atypia, areas of necrosis, a high rate of spindle cell proliferation, and the presence of mitotic figures indicate higher aggressiveness for the tumor (11). A 4/10 mitotic count per HPF and a Ki67 proliferation activity index of 30% were detected in our case. However, no necrosis or significant pleomorphism was observed. Because of high mitotic activity and a high Ki-67 proliferative index, our case was considered to have an aggressive clinical course and close clinical follow-up was recommended.

The treatment recommended for oral epithelioid hemangioendothelioma was extensive surgical resection followed by the regular follow-up (8).

In conclusion, only ten cases have been reported in the tongue in the literature. Our case was the eleventh case, and we aimed to report this case as a rare entity with an unusual location. A consensus on diagnosis may not be established by different pathologists in unusual cases. Therefore, it would be important to report the cases with rarely seen tumor localization in the literature.

## Conflict of Interest

The authors declare no conflict of interest.
